# Assessing Cyberbullying in Adolescence: New Evidence for the Spanish Version of the European Cyberbullying Intervention Project Questionnaire (ECIP-Q)

**DOI:** 10.3390/ijerph192114196

**Published:** 2022-10-30

**Authors:** Ildefonso Álvarez-Marín, Alicia Pérez-Albéniz, Beatriz Lucas-Molina, Vanesa Martínez-Valderrey, Eduardo Fonseca-Pedrero

**Affiliations:** 1 Faculty of Humanities and Social Sciences, Universidad Isabel I, 09003 Burgos, Spain; 2 Department of Educational Sciences, University of La Rioja, 26006 Logroño, Spain; 3 Department of Developmental and Educational Psychology, University of Valencia, 46010 Valencia, Spain; 4 Faculty of Education, University of Valladolid, 46010 Valencia, Spain

**Keywords:** ECIP-Q, cyberbullying, psychometric properties, measurement invariance, self-esteem, depression

## Abstract

The prevention of cyberbullying at school requires assessing its prevalence by means of brief measurement instruments with adequate psychometric properties. The present study aims to study the psychometric properties of the European Cyberbullying Intervention Project Questionnaire (ECIP-Q) in a sample of 1777 Spanish adolescents (54.1% women, *M* = 15.71 years; *SD* = 1.26), selected by stratified random cluster sampling. The two-factor model (victimization and aggression) displayed appropriate goodness of-fit indices. Configural measurement invariance model across gender was found. The omega reliability coefficient for the victimization subscale was 0.82, and for the aggression subscale was 0.68. The ECIP-Q scores were negatively associated with self-esteem and prosocial behavior, and positively associated with depression symptoms and emotional and behavioral difficulties. Significant differences were found between victim and non-victim groups, and between aggressor and non-aggressor groups on the same variables. Victims and aggressors scored lower on self-esteem, and higher on depression symptoms and emotional and behavioral difficulties than those not involved in cyberbullying situations. These findings contribute to demonstrate the satisfactory psychometric quality of the ECIP-Q scores as an assessment tool for cyberbullying in Spanish adolescents.

## 1. Introduction

Bullying is postulated as a problem that is present in schools and which generates social concern due to its negative physical and psychological effects, especially in adolescents between the ages of 14 and 18. In recent years, another form of peer harassment has emerged. This new form, known as cyberbullying or virtual bullying [1,2,3], occurs in the virtual scene, and therefore uses information and communication technologies (ICTs) as a tool to perpetuate this type of behavior. Despite the high number of studies focused on this phenomenon, certain discrepancies are still observed regarding the operational definition of cyberbullying [4,5,6]. One of the most used is the one that conceptualizes the phenomenon as bullying through ICT [7,8,9,10]. Although this is one of the most used definitions, it is convenient to differentiate between both types of peer-on-peer abuse. Bullying and cyberbullying coincide in many of their features, as well as in their associated consequences; however, the results obtained in previous works highlight the need to consider both forms of aggression as different phenomena with specific predictors and unique characteristics [11,12]. In the study carried out by Peter and Petermann [13], whose objective was to study the definitions of school cyberbullying formulated between 2012 and 2017, they evaluated constructs that were most repeated in the different conceptualizations of the phenomena. Within the definitions analyzed in the 24 documents that were part of the study, the descriptor «ICT» was collected 17 times, the notions of «intentionality» and «the objective is a person» were highlighted 15 times, “repetition” was mentioned 10 times, and the terms “harm”, “aggression”, and “imbalance of power” were pointed out 9, 8, and 4 times, respectively. All the previously mentioned descriptors, except the one referring to ICT, have frequently belonged to the definitions of bullying. However, due to the means by which it can be executed, school cyberbullying includes other characteristics such as the possibility of carrying out the abuse at any time, without the need for the victim to be present and consequently without a real sense of the damage caused, as well as the potential anonymity of the student aggressors [14,15], or the increased audience.

Some of the characteristics of bullying, such as the repetition of the aggression, present nuances when applied to cyberbullying. For example, it is not necessary for the aggressor to share a degrading photo of the victim more than once; uploading it one time already allows it to be observed and shared by a large group of viewers repeatedly [16]. As far as the power imbalance is concerned, it no longer has to be linked to the aggressor’s popularity or greater physical strength, all the aggressor needs is to have experience and skill in managing social networks [3,17]. The lack of a common and agreed definition for the phenomenon of school cyberbullying means that the items of the instruments developed for its measurement are heterogeneous and evaluate very different behaviors [18,19].

Different measurement instruments to analyze cyberbullying exist in the current literature [5,6,15,20,21,22,23]. However, the ECIP-Q, developed by Brighi et al. [24], stands out for being a brief instrument that structures the different forms of cyberbullying through 22 items (11 victimization and 11 aggression) [25]. Previous studies have confirmed the psychometric properties of the ECIP-Q scores [26,27,28,29,30,31]. The ECIP-Q’s items, which allow the operationalization of 11 behaviors related to cyberbullying, the briefness of its application, and the homogeneity of the results found when analyzing evidence of its validity and its internal consistency in the different studies allow the ECIP-Q to be considered an adequate instrument for measuring school cyberbullying [32]. However, it is still necessary to conduct new studies in representative samples of the population and test new statistical analyses (e.g., analysis of measurement invariance). In this sense, and given that it is difficult to find studies that use exactly the same instrument to assess school cyberbullying, it would be convenient to analyze the advantages and disadvantages of the existing tools before resorting to the development of new measurement instruments [5].

Furthermore, no research analyzing the relationship between profiles (victim or aggressor) evaluated using the ECIP-Q and other variables associated with cyberbullying has been found. Recent studies that use other questionnaires to evaluate cyberbullying show that both student victims [33,34,35,36] and aggressors [16,37,38] present symptoms of depression and antisocial behavior [39]. Some authors consider that school cyberbullying can generate a greater psychosocial maladjustment in the victims than that caused by face-to-face bullying [33,40] due to the aforementioned characteristics: large audience, anonymity of the bully, or the unlimited nature of the harassment [41]. Recent studies [42] that analyzed cyberbullying and neurocognition have shown the relationship between cyberbullying and social cognition and between being a cyberbully and having low empathy. Other research has revealed the effect of cognitive empathy on the behavior of adolescents involved in cyberbullying [43,44]. Cyberbullying can also cause a deterioration of self-esteem in the victim [45,46] and in the cyberbullying student [1,47], which can also lead to social rejection.

In this research context, the main goal of this study was to analyze the psychometric properties of the ECIP-Q scores in a representative sample of Spanish adolescents. From this general objective, the following specific objectives were derived (a) to study the internal structure of the ECIP-Q scores; (b) to determine the measurement invariance by gender; (c) to estimate the internal consistency of the ECIP-Q scores; (d) to analyze the relationship of the ECIP-Q scores with psychometric indicators of socio-emotional adjustment and mental health; and (e) to observe the difference in means of the ECIP-Q scores according to the profiles of the participants.

## 2. Materials and Methods

### 2.1. Sample and Procedure

Random sampling stratified by clusters was used for the selection of study participants. The population was composed of approximately fifteen thousand students from the Autonomous Community of La Rioja. The strata were created based on criteria such as school stage (Compulsory Secondary Education, High School, and Vocational Training), type of center (public or private-subsidized), and location (Rioja Baja, Rioja Media, and Rioja Alta). A total of 31 schools and 98 classrooms were selected for the study. The age of the 1972 students who answered the administered questionnaires ranged from 14 to 30 years. Students older than 19 years were excluded from the final sample. Participants with high scores (more than two points) on the revised Oviedo Infrequency Scale (INF-OV-R) [48] were also eliminated. The final sample consisted of 1777 students of Spanish nationality, 54.1% female, aged between 14 and 18 years (*M* = 15.71 years; *SD* = 1.26).

The battery of tests was administered collectively in groups of 15 to 25 students during regular class hours in a room specially prepared for it. A group of researchers trained in the protocols to be followed supervised the administration. The measuring instruments were administered by computer. Previously, the school management of the selected centers was contacted, and the families of the students were asked for their informed consent. Students were informed of the voluntary nature of their participation and the confidentiality of their answers. The study was approved by the Clinical Research Ethics Committee of La Rioja (CEICLAR).

### 2.2. Instruments

#### 2.2.1. European Cyberbullying Intervention Project Questionnaire (ECIP-Q [24]; Spanish Version Ortega-Ruiz et al. [32])

This questionnaire consists of 22 Likert-type items with five response options (0 = never, 1 = once or twice, 2 = once or twice a month, 3 = about once a week, 4 = more than once a week). The first eleven items evaluate behaviors related to victimization, while the next eleven measure different aggressive behaviors. To evaluate both dimensions, the items refer to actions such as saying bad words, excluding or spreading rumors, impersonating someone, etc., all carried out with technological tools and within a specific time interval [32]. Participants have to indicate the frequency with which they have experienced and/or participated in each of these situations. Adequate psychometric properties have been found in previous studies [26,49,50].

#### 2.2.2. Rosenberg Self-Esteem Scale (RSE [51]; Spanish Version Vázquez Morejón et al. [52])

This instrument allows self-esteem to be assessed through 10 items that are scored on a Likert-type scale (1 = totally disagree; 4 = totally agree). In this study, the Spanish version was used, which has shown adequate psychometric properties [52,53,54,55].

#### 2.2.3. Reynolds Adolescent Depression Scale-Short Form (RADS-SF [56]; Spanish Version Figueras-Masip et al. [57])

This self-report assesses the severity of depressive symptoms in adolescents. It consists of 10 items that are answered following a Likert-type scale with four response options (1 = almost never; 4 = almost always) which correspond to the four scales of the original version: anhedonia, somatic complaints, negative self-evaluation, and dysphoria. The RADS-SF has shown adequate psychometric properties in Spanish adolescents [58,59].

#### 2.2.4. Strengths and Difficulties Questionnaire (SDQ [60]; Spanish Version Ortuño-Sierra et al. [61])

This instrument evaluates behavioral and emotional difficulties and assesses social capacities. It consists of 25 items with a Likert-type response format of three options (0 = no, never, 1 = sometimes, 2 = yes, always) distributed in five subscales: emotional problems (SDQ-PREM), behavioral problems (SDQ-PRBE), problems with peers (SDQ-PRPE), hyperactivity (SDQ-HIP), and prosocial behavior (SDQ-PROS). The first four subscales make up the total behavioral and emotional difficulties score, where a higher score is indicative of poorer emotional and behavioral adjustment [62]. On the prosocial behavior subscale, a higher score indicates a higher social adjustment. The version adapted and validated for Spanish adolescents was used in this study [61,62].

#### 2.2.5. Oviedo Infrequency Scale Revised (INF-OV-R) [48,63,64]

This scale is used to detect random, pseudo-random, or dishonest responses. It is composed of 10 items that are answered with a Likert-type scale of five scores corresponding to categories (1 = completely disagree; 5 = completely agree). Students with two or more incorrect answers were removed from the sample. This scale has been shown to be effective in previous studies [48].

### 2.3. Data Analysis

First, the descriptive statistics of the ECIP-Q items were calculated for the sample. The analysis of the Mardia’s multivariate asymmetry skewness and kurtosis confirmed the breach of data normality.

Second, the internal structure of the ECIP-Q scores was examined using Confirmatory Factor Analysis (CFA). Guidelines for conducting factor analyses [65] were used. The hypothetical model of two related dimensions (victimization and aggression) [25] was tested. Based on the criteria of Hu and Bentler [66], the following indices were considered: Comparative Fit Index (CFI), General Fit Index (GFI), and Non-Normed Fit Index (NNFI) with values greater than 0.95; Root Mean Square Error Approximation (RMSEA) with values less than 0.05; Standardized Root Mean Square Residual (SRMR) with values less than 0.08. The Diagonally Weighted Least Squares (DWLS) estimation method was used due to failure of the multivariate normality assumption [67].

Third, an analysis of measurement invariance by gender was conducted. The different levels of invariance were contrasted by calculating the differences between the chi-square and CFI statistics, where differences in the CFI value of less than 0.01 indicate model fit [68].

Fourth, the McDonald’s omega coefficient, composite reliability index, and mean variance extracted were computed to estimate the reliability of the CEIP-Q scores.

Fifth, to obtain evidence of the relationship between the ECIP-Q scores and external variables, the association with different psychometric indicators of socio-emotional adjustment was analyzed using the Pearson coefficient. In the present study, participants of both sexes who had been the object of any of the 11 behaviors related to victimization and who had also not engaged in any cyberbullying behaviors with a minimum frequency of once or twice a month are identified as victims. Participants who indicated any of the 11 aggressive behaviors and who, in turn, had not suffered any cyberbullying behavior with a minimum frequency of once or twice a month were categorized as aggressors [23,24]. To wrap it up, the Student’s t-test was used for independent samples to check for statistically significant differences in the indicators of socio-emotional adjustment between the groups of victims and non-victims, aggressors and non-aggressors, and victims and aggressors. Robust tests were used when model assumptions were not met. To estimate the effect size, Cohen’s “d” statistic was used.

The statistical programs SPSS (IBM Statistical Package for the Social Sciences, Armonk, New York, United States of America) version 24) and JASP (Jeffrey’s Amazing Statistics Program, Amsterdam, The Netherlands) version 0.16, were used.

## 3. Results

### 3.1. Descriptive Statistics of the ECIP-Q

Table 1 presents the statements of the 22 items of the questionnaire with its descriptive statistics and the reliability indicators of the scores for each subscale. The items with the highest mean values were those which asked about having been insulted or having insulted other people through email or messaging (items V1 and A12). Items that referred to the same behavior, but through other people, also reached the highest averages (items V2 and A13). The discrimination indices of the items were higher than 0.30, with the exception of three items.

### 3.2. Validity Evidence Based on Internal Structure

The goodness-of-fit indices for the two-factor model were: CFI = 0.96, GFI = 0.98, NNFI = 0.95, RMSEA = 0.051, and SRMR = 0.13 [68,69,70]. The goodness-of-fit indices for the one-dimensional model were: CFI = 0.94, GFI = 0.98, RMSEA = 0.061, and SRMR = 0.15. The fully standardized factor loadings for the bidimensional model are shown in Table 2. All estimated factor loadings were greater than 0.50 and statistically significant (*p* < 0.001).

### 3.3. Measurement Invariance of the ECIP-Q’ Scores by Gender

Regarding the study of measurement invariance according to gender, the configural model showed the following fit indices: χ^2^/gl = 1.18, CFI = 0.96, TLI = 0.95 and RMSEA = 0.01. In the metric invariance model, the indices presented a lower fit: χ^2^/gl = 1.73, CFI = 0.83, TLI = 0.82 and RMSEA = 0.03, with respect to the base model. The differences between the models’ values (ΔSB-χ^2^ = 262.51; ΔCFI = 0.13) report on the convenience of applying the configural invariance model and not continuing to test more restrictive models.

### 3.4. Study of the Reliability of the ECIP-Q’ Scores

The omega coefficient, the composite reliability index and the values of the mean variance extracted confirmed the reliability of the subscales are shown in Table 1. The omega reliability coefficient for the victimization subscale was 0.82 and for the aggression subscale was 0.68.

### 3.5. Validity Evidence of Relationships with Other External Variables

Table 3 shows the Pearson correlations between the measurement instruments administered. The victimization subscale statistically and significantly correlated with all the variables, negatively with self-esteem and SDQ prosocial behavior, and positively with symptoms of depression and the SDQ subscales. The same pattern of results was found for the aggression subscale, except for the emotional problems subscale of the SDQ, whose correlation was not statistically significant.

To analyze the differences in means according to the profiles (victim and aggressor) of cyberbullying in self-esteem, symptoms of depression, and emotional and behavioral difficulties, the means obtained in the ECIP-Q were contrasted in three groups of pairs: (1) the victim and non-victim students, (2) the aggressor and non-aggressor students; and, finally, and (3) the victim and aggressor students. The results are presented in Table 4.

The statistically significant differences found are the following: The students who were victims of school cyberbullying obtained higher scores in the depression scale and in emotional problems, behavioral problems, relationship with their peers, and hyperactivity, obtaining lower scores in self-esteem. When contrasting the group of aggressors with that of non-aggressors, the former achieved higher scores in depression, and emotional and behavioral problems, and lower scores in self-esteem and prosocial behavior. The differences between the student victim and aggressor were statistically significant in the overall score on the SDQ scale, in behavioral problems and in prosocial behaviors, always in favor of the victims. A moderate effect size was obtained in all the differences that were significant.

## 4. Discussion

The objective of this study was to analyze the psychometric properties of the ECIP-Q scores in a representative sample of Spanish adolescents. For this purpose, the reliability of its scores, the internal structure, measurement invariance by gender, the relationship of the ECIP-Q scores with other psychometric indicators of psychological adjustment, and the difference in means of the ECIP-Q scores according to participant profiles were evaluated. Having screening instruments with adequate psychometric quality to analyze this type of phenomenon during adolescence is especially relevant, given that it is an important stage of human development, and it will allow implementing prevention strategies to minimize the personal, family, and social impact of problems associated with school bullying and cyberbullying [71].

The analysis of the psychometric properties of the ECIP-Q scores provides new validity evidence and reliability of the scores in a large random sample of Spanish adolescents derived from the general population. As in previous studies [26,30,32], the ECIP-Q has a two-factor structure, the first eleven items are grouped in the victimization factor and the next eleven in the aggression factor; the reliability of the ECIP-Q subscale scores can be considered satisfactory [72]. All of the factor fit indices reach optimal values, except for the SRMR index, which did not reach the expected value, perhaps because of its sensitivity to the violation of the normality of the data. The configural invariance according to the gender has been confirmed, an issue not analyzed in previous studies, which implies that the factorial structure found is similar in both groups [73].

With regard to the study of evidence with other variables, the ECIP-Q revealed positive and statistically significant associations with variables related to mental health such as emotional difficulties, behavioral problems, and depressive symptoms. In particular, both victimization and aggression subscales were negatively related with lower self-esteem and prosocial behavior and positively related with self-reported depression symptoms and behavioral and emotional difficulties. Similar results were reported in previous studies [74,75]. In addition, higher scores on the victimization factor were significantly related to higher values on the SDQ subscale that measures emotional problems, which is not the case with scores on the aggression factor.

When contrasting the means of the groups in each of the external variables (i.e., victims vs. non-victims, aggressors vs. non-aggressors, victims vs. aggressors), the adolescent victims of cyberbullying [76,77] and their aggressors present lower levels of self-esteem [75,78] and greater depressive symptomatology than those who are not involved in this type of behavior [74,79,80], which could be indicating that cyberbullying is a factor that worsens depression, self-image, and general self-confidence both in those who suffer from it and in the people who exercise it [81]. These results coincide with those obtained in other studies of the reviewed literature [82,83,84,85,86]. This idea is confirmed, given that no significant differences were found between the group of victims and the group of aggressors in terms of self-esteem or depression. In the case of behavioral and emotional difficulties, they are also more pronounced in the aggressors and in the victims than in the students who do not participate in schoolq cyberbullying [87]; the victims also present more problems in their social relationships compared to their aggressors. These results coincide with those found in other studies [88,89,90] that indicate that cybervictims and cyberbullies show less social competence. Other investigations that use the SDQ, developed by Sidera et al. [49] and by Longobardi et al. [37], confirm the relationship between being a victim of school cyberbullying and suffering emotional and behavioral problems. Through the SDQ, Kaiser et al. [91] also found that cybervictims present more emotional and behavioral problems than non-victims, and that cyberbullies show greater prosocial behavior than victims.

The present study meets the objective of providing new evidence about the psychometric quality of the ECIP-Q scores in non-clinical adolescents. The present work provides valuable information for the screening of cyberbullying in adolescent populations in order to evaluating the prevalence of cyberbullying, and its potential negative consequences for school settings [92,93]. This type of evaluation is essential for psychological interventions that aim to discourage its appearance. Recent meta-analyses conducted by Hannah Gaffney et al. [94,95,96,97] show that prevention programs can reduce bullying and cyberbullying levels in schools by up to 20%, and that there are several components that appear to be more effective than others. These components include adopting a whole-school approach to prevention that involves the entire school community, providing teacher training, implementing classroom activities based on cooperative learning, encouraging peer support, holding meetings with families, providing increased supervision in high-risk areas of the school (e.g., school yard and corridors), and establishing clear disciplinary measures against bullying and cyberbullying, among others.

The present study is not exempt from some limitations. In the first place, self-report type instruments have been used in this study with the common limitations. Second, despite having worked with a large and probabilistically selected sample, it is limited to a single Spanish autonomous community, La Rioja. Third, it is a cross-sectional study, so care must be taken when establishing cause–effect relationships.

## 5. Conclusions

The results from this study allow new research to compare the prevalence of both virtual and face-to-face bullying, so that the relationship between both forms of abuse, its evolution and risk factors, and the mediating variables involved can be analyzed [46,98]. The verification of the existence of dangers inherent in the use of ICTs, which is increasingly widespread during childhood and adolescence [99], reinforces the argument on the importance of conducting studies and designing psychological interventions in schools in order to prevent the appearance of these behaviors, develop action protocols when they have occurred, and mitigate the possible negative consequences derived from the phenomenon [100]

## Figures and Tables

**Table 1:** Reliability of the subscale scores of the European Cyberbullying Intervention Project Questionnaire and descriptive statistics of the items.

ECIP-Q	Omega	CR	MVE	Item		*M*	*SD*	Symmetry	Kurtosis	DI
Cybervictimization Subscale	0.82	0.94	0.59	1	Someone said nasty things to me or called me names using texts or online messages.	0.32	0.70	2.66	7.96	0.54
				2	Someone said nasty things about me to others either online or through text messages.	0.26	0.63	2.92	9.65	0.63
				3	Someone threatened me through texts or online messages.	0.10	0.41	5.33	34.80	0.59
				4	Someone hacked into my account and stole personal information.	0.04	0.24	8.90	106.89	0.52
				5	Someone hacked into my account and pretended to be me.	0.05	0.25	7.75	83.35	0.50
				6	Someone created a fake account, pretending to be me.	0.05	0.26	6.15	52.08	0.44
				7	Someone posted personal information about me online.	0.04	0.26	7.46	69.95	0.53
				8	Someone posted embarrassing videos or pictures of me online.	0.04	0.26	8.85	96.81	0.27
				9	Someone altered pictures or videos of me that I had posted online	0.05	0.27	7.80	79.10	0.35
				10	I was excluded or ignored by others in a social networking site or Internet chatroom.	0.10	0.41	5.41	36.30	0.46
				11	Someone spread rumors about me on the Internet	0.11	0.45	5.34	34.44	0.63
Cyberaggression Subscale	0.68	0.94	0.58	12	I said nasty things to someone or called them names using texts or online messages].	0.19	0.55	3.62	15.48	0.50
				13	I said nasty things about someone to other people either online or through text messages.	0.19	0.54	3.54	14.67	0.55
				14	I threatened someone through texts or online messages.	0.06	0.32	7.01	60.64	0.44
				15	I hacked into someone’s account and stole personal information.	0.02	0.18	12.83	197.96	0.50
				16	I hacked into someone’s account and pretended to be them.	0.02	0.18	14.06	233.41	0.49
				17	I created a fake account, pretending to be someone else.	0.05	0.26	6.74	63.59	0.23
				18	I posted personal information about someone online.	0.02	0.19	14.77	246.82	0.28
				19	I posted embarrassing videos or pictures of someone online	0.03	0.22	11.35	155.68	0.36
				20	I altered pictures or videos of another person that had been posted online.	0.05	0.27	7.21	64.43	0.34
				21	I excluded or ignored someone in a social networking site or Internet chatroom	0.09	0.38	5.40	36.30	0.31
				22	I spread rumors about someone on the Internet	0.03	0.23	8.81	96.16	0.44

Note: ECIP-Q: European Cyberbullying Intervention Project Questionnaire; CR: Composite reliability; MVE: Mean variance extracted; DI: Discrimination index.

**Table 2:** Standardized factor loadings for the two-factor model of the European Cyberbullying Intervention Project Questionnaire.

					Confidence Interval (95%)	
Factor	Item	Estimated Factor Loading	Typical Error	Z Statistic	Lower Limit	Upper Limit
I	1	0.81	0.01	63.81	0.79	0.83
	2	0.86	0.01	67.94	0.84	0.88
	3	0.78	0.02	50.59	0.75	0.81
	4	0.82	0.02	45.07	0.78	0.86
	5	0.79	0.02	44.88	0.76	0.82
	6	0.69	0.02	36.41	0.65	0.73
	7	0.79	0.02	42.82	0.76	0.82
	8	0.72	0.02	35.57	0.68	0.76
	9	0.65	0.02	32.29	0.61	0.69
	10	0.64	0.02	36.19	0.61	0.67
	11	0.84	0.02	56.14	0.82	0.86
II	12	0.87	0.02	58.83	0.84	0.90
	13	0.83	0.02	56.75	0.80	0.86
	14	0.78	0.02	40.41	0.74	0.82
	15	0.92	0.02	42.07	0.88	0.96
	16	0.88	0.02	41.09	0.84	0.92
	17	0.54	0.02	24.96	0.50	0.58
	18	0.78	0.03	28.12	0.73	0.83
	19	0.72	0.03	28.12	0.67	0.77
	20	0.60	0.02	28.47	0.56	0.64
	21	0.59	0.02	29.05	0.55	0.63
	22	0.76	0.02	35.72	0.72	0.80

**Table 3:** Pearson correlations between the scores of the European Cyberbullying Intervention Project Questionnaire and different indicators of socio-emotional and behavioral adjustment.

Variable.	ECIP-Q Victimization	ECIP-Q Aggression	RSE	RADS	SDQ	SDQ-PREM	SDQ-PRCD	SDQ-PRCM	SDQ-HIP
ECIP-Q Aggression	0.43 **								
RSE	−0.19 **	−0.06 *							
RADS	0.27 **	0.14 **	−0.73 **						
Total SDQ	0.22 **	0.11 **	−0.58 **	0.68 **					
SDQ-PREM	0.12 **	0.02	−0.61 **	0.64 **	0.73 **				
SDQ-PRBE	0.17 **	0.16 **	−0.22 **	0.36 **	0.64 **	0.192 **			
SDQ-PRPE	0.24 **	0.06 *	−0.36 **	0.50 **	0.55 **	0.35 **	0.20 **		
SDQ-HIP	0.10 **	0.08 **	−0.26 **	0.27 **	0.68 **	0.23 **	0.44 **	0.05 *	
SDQ-PROS	−0.06 *	−0.12 **	0.10 ***	−0.21 **	−0.18 **	−0.03	−0.28 **	−0.15 **	−0.14 **

Note: ECIP-Q = European Cyberbullying Intervention Project Questionnaire; RSE = Rosenberg Self-Esteem Scale; SDQ = Strengths and Difficulties Questionnaire, total difficulties score; SDQ-PREM = Emotional problems; SDQ-PRBE = Behavioral problems; SDQ-PRPE = Peer Problems; SDQ-HIP = Hyperactivity; SDQ-PROS = Prosocial behavior. * *p* ≤ 0.05, ** *p* ≤ 0.01.

**Table 4:** Contrasts of means between groups of victims and aggressors of the European Cyberbullying Intervention Project Questionnaire.

Cluster	Victim		Non-Victim				Aggressor		Non-Aggressor				Victim		Aggressor			
Variable	*M*	*SD*	*M*	*SD*	T	d	*M*	*SD*	*M*	*SD*	t	d	*M*	*SD*	*M*	*SD*	t	d
RSE	27.65	6.83	31.08	5.36	−5.32 *	−0.56	29.30	6.30	30.92	5.49	−2.47 *	0.29	27.65	6.83	29.30	6.30	−1.70	−0.25
RADS-SF	19.25	6.22	16.16	4.21	5.29 *	0.58	18.72	5.54	16.26	4.36	3.77 *	−0.49	19.25	6.22	18.72	5.54	0.60	0.09
Total SDQ	14.62	6.14	10.69	4.92	6.77 *	0.71	12.65	4.93	10.87	5.09	2.94 *	−0.35	14.62	6.14	12.65	4.93	2.32 *	0.34
SDQ-PREM	4.49	2.81	3.35	2.35	4.25 *	0.44	3.82	2.52	3.41	2.40	1.45	−0.17	4.49	2.81	3.82	2.52	1.65	0.24
SDQ-PRBE	2.28	1.77	1.70	1.52	3.45 *	0.35	2.36	1.84	1.70	1.53	3.04 *	−0.39	2.27	1.77	2.36	1.84	−0.34	−0.05
SDQ-PRPE	2.74	2.13	1.34	1.50	7.00 *	0.76	1.70	1.79	1.42	1.55	1.55	−0.18	2.74	2.13	1.70	1.79	3.47 *	0.52
SDQ-HIP	5.12	2.25	4.30	2.15	3.96 *	0.38	4.76	2.18	4.34	2.16	1.62	−0.19	5.12	2.25	4.76	2.18	1.10	0.16
SDQ-PROS	8.50	1.48	8.57	1.41	−0.46	−0.045	8.03	1.59	8.59	1.41	−3.33 *	0.40	8.50	1.48	8.03	1.59	2.11 *	0.31

Note: M = Mean; SD = Standard deviation; ECIP-Q = European Cyberbullying Intervention Project Questionnaire; RSE = Rosenberg Self-Esteem Scale; Total SDQ = Strengths and Difficulties Questionnaire, total difficulty score; SDQ-PREM = Emotional problems; SDQ-PRBE = Behavioral problems; SDQ-PRPE = Peer problems; SDQ-HIP = Hyperactivity; SDQ-PROS = Prosocial behavior. * *p* ≤ 0.05.

## Data Availability

Not appliable.
